# Supplementary material for: Development of a computer-aided design software for the quantitative evaluation of aesthetic damage
Source: PLoS One. 2019 Dec 18;14(12):e0226322. doi: 10.1371/journal.pone.0226322 (PMC6919621; doi:10.1371/journal.pone.0226322)
Supplement: S1 Code site — 1. (ZIP) [file pone.0226322.s001.zip › [EESC_jr][Projeto]Relatorio_07_21/reports/templates_zip/gentelella-master/vendors/morris.js/examples/donut-formatter.html]

# Donut Chart

```
Morris.Donut({
  element: 'graph',
  data: [
    {value: 70, label: 'foo', formatted: 'at least 70%' },
    {value: 15, label: 'bar', formatted: 'approx. 15%' },
    {value: 10, label: 'baz', formatted: 'approx. 10%' },
    {value: 5, label: 'A really really long label', formatted: 'at most 5%' }
  ],
  formatter: function (x, data) { return data.formatted; }
});
```
